# Irradiating the Path to High‐Efficiency Zn‐Ion Batteries: An Electrochemical Analysis of Laser‐Modified Anodes

**DOI:** 10.1002/gch2.202400105

**Published:** 2024-08-29

**Authors:** Ramona Durena, Leonid Fedorenko, Nikita Griscenko, Martins Vanags, Liga Orlova, Pavels Onufrijevs, Sandra Stanionyte, Tadas Malinauskas, Anzelms Zukuls

**Affiliations:** ^1^ Institute of Materials and Surface Engineering Faculty of Natural Sciences and Technology Riga Technical University Paula Valdena Street 7 Riga LV‐1048 Latvia; ^2^ Lashkaryov Institute of Semiconductor Physics National Academy of Sciences of Ukraine Prospect Nauki 41 Kyiv 03028 Ukraine; ^3^ Institute of Technical Physics Faculty of Natural Sciences and Technology Riga Technical University Paula Valdena Street 7 Riga LV‐1048 Latvia; ^4^ Center for Physical Sciences and Technology Sauletekio Ave. 3 Vilnius LT‐10257 Lithuania; ^5^ Institute of Photonics and Nanotechnology Vilnius University Sauletekio Ave. 3 Vilnius LT‐10257 Lithuania

**Keywords:** Cyclic voltammetry, energy storage, Impedance spectroscopy, Laser irradiation, Raman spectroscopy, Surface morphology, Zn‐ion batteries (ZIBs)

## Abstract

Global energy consumption is increasing yearly, yet the world is trying to move toward carbon neutrality to mitigate global warming. More research is being done on energy storage devices to advance these efforts. One well‐known and widely studied technology is Zn‐ion batteries (ZIBs). Therefore, this paper demonstrates how laser irradiation at wavelengths of 266 and 1064 nm, in the presence of air or water, can enhance the electrochemical performance of metallic zinc anode in alkaline electrolyte. The obtained samples are characterized using X‐ray diffraction analysis, scanning electron microscopy, and Raman spectroscopy. Then, the electrochemical properties are studied by cyclic voltammetry and impedance measurements. Results indicate that the laser processing of the Zn sample increases surface‐specific capacity by up to 30% compared to the non‐irradiated Zn sample. Furthermore, electrochemical measurements reveal enhanced participation of metallic Zn grains in the oxidation and reduction processes in irradiated samples. In future research, integrating laser treatment into electrode preparation processes can become essential for optimizing anode battery materials.

## Graphical abstract

The improvement of battery electrodes is very important for high‐performance products. Laser treatment of zinc anode materials allows for improving the surface‐specific capacity of the electrode by up to 30% compared to an un‐irradiated zinc electrode. Anode surface laser treatment in combination with other electrode improvement methods opens up the possibility of creating efficient water‐based Zn ion batteries.

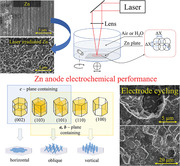

## 1 Introduction

Global energy production from renewable sources continues to increase alongside that from fossil fuels. Moreover, global energy consumption increases annually. Achieving carbon neutrality necessitates reducing fossil fuel consumption while scaling up renewable energy production.^[^ ^1^ ^]^ A significant hurdle for the widespread adoption of renewable energy is the variability in energy production over time. Implementing diverse energy storage systems is crucial to store surplus energy for future use.^[^ ^2^, ^3^, ^4^ ^]^

For now, the most used secondary battery systems are Li‐ion batteries (f). They have been and will probably continue to dominate the portable device market. However, LIBs are not economically viable for battery energy storage systems due to the scarcity of the necessary metals, and the safety risks the LIBs oppose.^[^ ^5^, ^6^, ^7^, ^8^, ^9^, ^10^ ^]^ Alternative to LIBs for larger energy storage facilities are Zn‐ion batteries (ZIBs). Metallic Zn is more readily available, with lower costs and reduced safety risks.^[^ ^11^, ^12^, ^13^ ^]^ However, applying metallic Zn as an anode has several solvable drawbacks. The main problems with Zn anodes are electrochemical hydrogen evolution reaction (HER), corrosion passivation, and dendrite growth.^[^ ^14^, ^15^, ^16^ ^]^

HER is a severe ZIB problem because hydrogen gas generation can lead to battery swelling or even explosion. Theoretically, HER is more thermodynamically favorable than Zn plating because its standard potential is lower. In neutral electrolytes, the equilibrium potential of Zn^2+^/Zn is −0.76 V versus standard hydrogen electrode (SHE), and H_2_O/H_2_ is 0 V versus SHE; however, in alkaline electrolytes, the equilibrium potentials are −1.26 V versus SHE and −0.83 V versus SHE, respectively.^[^ ^17^, ^18^, ^19^, ^20^ ^]^ Although HER is theoretically more favorable, the Zn plating reaction will overtake HER due to the low hydrogen ion activity and high overpotential. However, HER can still occur under certain conditions like high polarization during charging, high current density, and low potential. As a result, the Coulombic efficiency (CE) decreases, hazardous hydrogen gas is released, the pH of the electrolyte changes locally, the amount of electrolyte decreases, and the battery dries out over a more extended period of time.^[^ ^21^, ^22^, ^23^, ^24^, ^25^, ^26^ ^]^

Similar to the electrochemical HER process, a chemical reaction of Zn metal with an aqueous electrolyte can also occur, resulting in the release of gaseous hydrogen and the conversion of Zn from the metal to the solution in the form of the Zn^2+^ ion, thereby losing capacity. In addition to this corrosion process, OH^−^ ions are formed due to water splitting, which increases the pH of the electrolyte. Thus, inert by‐products are formed because of these pH changes, which cover the surface of the anode and interfere with the further progress of the desired reaction. However, these products are not dense enough to stop the progress of side reactions.^[^ ^27^, ^28^, ^29^, ^30^, ^31^ ^]^

The formation of dendrites in batteries on the anode surface is also common in other metal anode batteries, such as LIBs.^[^ ^32^, ^33^ ^]^ The pre‐conditions for their formation are widely studied and well understood. The leading cause of their formation is related to inhomogeneities of the anode surface, such as protrusions, crystallite boundaries, lattice defects, impurities, etc., which lead to an inhomogeneous electric field. This, in turn, promotes inhomogeneous deposition of Zn, forming peaks, which further contribute to various “tip” effects. Such uncontrolled growth of dendrites usually leads to separator piercing, short‐circuiting, or “dead” zinc in case of dendrite breakage, which reduces anode capacity and CE.^[^ ^34^, ^35^, ^36^, ^37^ ^]^ According to the literature, the Zn (002) plane is the most suitable for battery anodes compared to Zn (100) and Zn (101) growth planes.^[^ ^38^ ^]^ The Zn (002) plane has a smaller self‐diffusion barrier, resulting in lower resistance to ad‐atom movement on the surface of Zn. Limited ad‐atom movement is the main reason for dendrite growth on Zn (100) and Zn (101) planes. The growth and dissolution of Zn/Zn^2+^ on a polycrystalline Zn surface lead to a loose and porous deposition of the Zn layer, which negatively impacts the reversibility of the anode. Additionally, side reactions, by‐product accumulation, and hydrogen evolution are more pronounced on these surfaces.^[^ ^39^ ^]^

All these processes are interrelated and reduce the capacity and efficiency of the Zn anode. The main directions as the scientific community tries to prevent these unwanted actions and improve the Zn anode performance are coating the electrode or modifying the electrolyte that controls and guides the stripping/plating reaction. Some coating options include CaCO_3_,^[^ ^40^ ^]^ ZnO,^[^ ^41^, ^42^, ^43^ ^]^ ZrO_2_^[^ ^44^, ^45^, ^46^ ^]^ and TiO_2_.^[^ ^47^, ^48^, ^49^ ^]^ Other scientists have used a slightly different approach to coating Zn anode by employing different polymer layers.^[^ ^50^, ^51^, ^52^, ^53^ ^]^

The roll‐press formation of Zn sheets induces surface defects such as roughened surfaces, scratches, folds, and new edges that are prone to dendrite growth.^[^ ^39^ ^]^ During the manufacturing process of roll‐pressed Zn, the (002) textured plane structure formation can be promoted through heating‐rolling processes. However, the previously mentioned defects still occur.^[^ ^39^ ^]^ To eliminate the effects of introduced surface defects, surface polishing can be utilized. Studies show that polishing can increase the cycling lifespan of an electrode more than seven times.^[^ ^39^ ^]^ Various methods can be used for metal surface polishing, including mechanical,^[^ ^39^ ^]^ chemical,^[^ ^54^ ^]^ electrical^[^ ^55^ ^]^ and laser^[^ ^56^ ^]^ polishing.

Laser processing is cost‐efficient and compatible for large‐scale production compared to various chemical routes.^[^ ^57^ ^]^ However, this approach has received very little attention regarding Zn anode as only a few researchers have published work in this area. C. Yang et al.^[^ ^58^ ^]^ have modified the Zn anode with a laser‐induced graphene coating, Z. Na et al.^[^ ^59^ ^]^ have used a laser lithography strategy, D. Yao et al.^[^ ^60^ ^]^ have applied femtosecond‐laser filament texturing, and H. Jin et al.^[^ ^61^ ^]^ have employed the surface texture through a laser‐micromachining method. All these works have presented effective strategies to improve the Zn anode, highlighting the need for more attention to the laser pretreatment of metal anodes. This approach could be particularly beneficial for roll‐pressed Zn substrates, as it can address surface defects and enhance Zn growth and dissolution properties. By reducing the residual stresses formed during the manufacturing of Zn sheets, oriented growth of newly plated Zn metal layers can be expected. Also, by combining it with separators, it would be possible to provide the desired uniform hexagonal zinc deposition.^[^ ^62^ ^]^ Therefore, in this study, the Zn anode surface was modified using pulsed laser radiation. It is well known that the effective surface area of the working electrode significantly influences charge accumulation and current flow in the anode‐electrolyte system. Pulsed laser radiation, which reaches or exceeds the microablation threshold in terms of energy density (fluence ─ F), is recognized as highly efficient for modifying this parameter. The aim of this work was to investigate how laser processing with varying energy densities and different environments (air, water) in the active technological zone influences structural properties. Additionally, the electrochemical properties of the half‐cell system consisting of a zinc plate anode and a 1 m KOH electrolyte solution were examined. This investigation revealed a surface‐specific capacity increase of up to 30%, decreased charge transfer resistance, and more pronounced Zn crystal grain boundaries after electrochemical cycling.

## 2 Experimental Section

Zinc plates were purchased from Goodfellow Cambridge Ltd. (Zinc foil, 0.20 mm, 99.95+%), deionized waters was prepared using Adrona Crystal Sterifeed and used without further purification, KOH (pallets for analysis) was purchased from Sigma Aldrich and used without further purification. The zinc plate was washed/rinsed in deionized water/ethanol before laser irradiation to remove loose metal particles and degrease them from factory oils.

**Figure 1:**
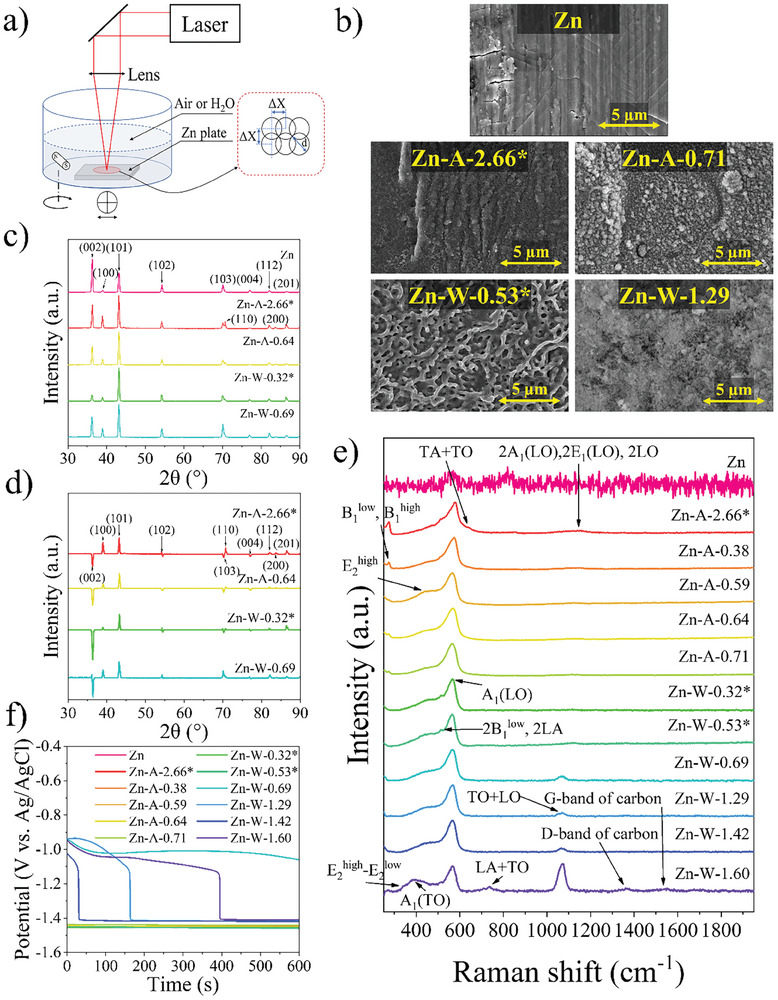
Pure and irradiated Zn sample characterization as prepared before electrochemical measurements: a) sample irradiation setup; b) SEM images; c) XRD diffractograms; d) difference between sample XRD and pure Zn (sample XRD spectrum minus pure Zn XRD spectrum); e) Raman analysis; f) OCP.

Zinc metal plates were irradiated with a nanosecond pulse Nd:YAG laser (model: NL301G, produced by Ekspla, Lithuania). Laser processing involved two different wavelengths (λ_1_ = 1064 and λ_2_ = 266 nm), with fluences ranging from 0.32 to 2.66 J cm^−2^, under various environmental conditions (air and deionized water). The Zn surface was irradiated using pulses with a duration of t_p_ = 6 ns in scanning mode, with a repetition frequency of laser pulses in the range from 1 to 10 Hz. This setup allowed control over the overlap of the laser spot on the Zn plate surface. A schematic of the irradiation process is shown in **Figure** **1**a, where ΔX = 0.125 mm and d = 1.2 mm. Additionally, the laser wavelengths were varied, considering the different interactions of UV (266 nm) and infrared (IR) (1064 nm) rays with zinc oxide (ZnO) that may form during laser processing. The scanning step was selected close to the diameter of the laser spot to maximize surface heterogeneity and effective area. Detailed information about the irradiated samples can be found in Table S1 (Supporting Information).

The structural characterization of the samples before and after electrochemical measurements was performed using X‐ray diffraction (XRD). Measurements were performed using the Smartlab (Rigaku, Japan) diffractometer with a 9 kW rotating Cu anode X‐ray generator. Theta/2theta scans were measured with a step size of 0.02°, with a total scan range from 30 to 90°. Obtained XRD results were analyzed using the PDF 4+ database.

Raman spectra analysis was performed for the samples before and after CV measurements at room temperature under 500x magnification using a Renishaw In‐ViaV727 spectrometer in backscattering geometry. The spectrometer was operated with an Ar^+^ green laser (wavelength λ = 514.5 nm, grating −1200 mm^−1^, power −10 mW), exposure time 10 s.

FEI Nova SEM230 and Hitachi TM3000 Tabletop scanning electron microscope (SEM) with an acceleration voltage of 10 kV and 15 kV were used to characterize sample surface morphology.

Total reflectance spectra were measured with a UV‐Vis‐NIR optical spectrometer (Ossila Optical Spectrometer). The measurement system consisted of a light source Ocean Insight “Broadband LED” and integrated sphere (Thorlabs, General‐Purpose Ø50 mm Integrating Spheres).

Various electrochemical properties were measured using Potentiostat‐Galvanostat Autolab PGSTAT302N. Cyclic voltammetry (CV) measurements were performed in the “TSC Surface” (from rhd instruments) measuring cell using a 3‐electrode system configuration. As received and laser irradiated sample zinc plates were used as working electrodes, 1 mL of 1 m KOH solution was used as an electrolyte, a platinum rod was used as a counter electrode, and a double junction configuration Ag/AgCl (3 m KCl) electrode was used as a reference electrode. For this study, no separators were used, to exclude the interference of the separator‐induced growth effects on the Zn anode that has been mentioned in literature.^[^ ^62^ ^]^ The CV measurements were performed in a potential window of −1.9 V to −0.6 V Ag AgCl^−1^ with variable scan rates from 0.005 to 0.1 V s^−1^. Impedance spectroscopy (EIS) was performed by applying a +/−150 mV small perturbation voltage in the 100 kHz to 0.1 Hz frequency range. For obtained data and equivalent scheme analysis, NOVA 2.1 software was used.

## 3 Results and Discussion

### 3.1 Sample Characterization

The obtained sample abbreviations are based on the applied fluence and their irradiation parameters, and visual photos are shown in Table S1 (Supporting Information). Visual observations concluded that laser‐irradiated sample surface morphology is homogeneous throughout all the irradiated 12 × 12 mm squares. Changes in the surface morphology and color were noticed. Diffuse reflectance measurements were performed to evaluate optical changes (Figure S1, Supporting Information) in the laser‐irradiated samples. The obtained spectra show that the surface of the irradiated samples reflects less light than a pure Zn plate. This observation can be attributed to the formation of a ZnO layer on the irradiated surfaces. Similar findings of ZnO formation have also been observed when irradiating Zn samples in air and ethanol medium.^[^ ^63^ ^]^ The resulting ZnO layer may contain interstitials and crystal defects, which would explain the reduced relative diffuse reflectance. Additionally, possible signs of luminescence can be observed in the 350–400 nm range, further indicating ZnO formation.^[^ ^64^ ^]^

Surface imaging was performed using SEM to investigate the obtained samples further. The SEM images are shown in Figure 1b (images for all samples are shown in Figure S2, Supporting Information). Characteristic signs of cold metal rolling can be observed on the surface of the non‐irradiated sample. The surface of a Zn plate consists of pressed‐down sharp‐edged grains with visible stretching marks. However, following laser irradiation, the Zn surface melted to varying degrees, which, depending on the applied laser power and irradiation environment, affected either shallower or deeper surface layers. The most pronounced Zn surface melting occurred in samples irradiated in air at a wavelength of 1064 nm. As a result, droplet‐like frozen structures formed on the surface of all these samples. A similar melting effect of laser‐irradiated Zn has also been observed in the literature.^[^ ^65^ ^]^ In contrast, samples irradiated with a 1064 nm laser in an aqueous environment formed loose ZnO structures, beneath which a molten layer of uniformly frozen globular droplets developed. However, samples irradiated in air using a 266 nm laser melted more uniformly and had smoother surfaces without droplet‐like structures. These samples were more similar to the unmodified Zn substrate with slight melting features, whereas samples irradiated in a water medium developed a crinkled melt structure.

The XRD measurements (Theta/2theta) for samples after laser irradiation were performed, to observe the changes in the crystallinity of the Zn surfaces perpendicularly to the growth direction (Figure 1c). For better comparison, the obtained spectra were normalized by their intensity. The diffractograms show that the Zn samples consist of a hexagonal Zn phase with peaks (PDF Card No.: 00‐004‐0831) located at 2θ = 36.5, 39.2, 42.5, 54.5, 70.3, 71.0, 77.3, 82.1, 83.7, and 86.5° corresponding to *hkl* of atomic planes (002), (100), (101), (102), (103), (110), (004), (112), (200), and (201).^[^ ^66^, ^67^ ^]^ The XRD results of the pristine Zn plate show a typical pattern for a polycrystalline Zn plate, with a dominant (002) basal plane that is parallel to the sheet surface due to rolling deformations induced during manufacturing.^[^ ^68^ ^]^ Laser treatment reduces the dominance of the (002) basal plane by re‐melting the surface and revealing an underlying polycrystalline structure dominated by the (101) plane. Figure 1d, where the pure Zn substrate spectrum is subtracted from the irradiated sample spectra, shows the most noticeable changes in polycrystallinity due to the multidirectionally oriented hexagonal structure of Zn. Unfortunately, SEM images of the irradiated samples (Figure 1b) only reveal the visible melt structures, with the polycrystalline structure lying beneath the melted layer.

To obtain more information about the surface, Raman analysis was performed. The results are shown in Figure 1e. The structure of hexagonal wurtzite ZnO can be distinguished in Raman measurements by the first‐order optical phonon modes. For perfect wurtzite ZnO crystals, optical phonon (Γ_opt_) modes can be described by Γ_opt_ = 1A_1_ + 2B_1_ + 1E_1_ + 2E_2_.^[^ ^69^ ^]^ All visible phonon modes for Zn samples are summarized in **Table** **1**. Observations point to hexagonal wurtzite structure ZnO growth with a space group of *P*63*mc*. The visible ZnO maxima at 382, 441, and 585 cm^−1^ correspond to the polar A_1_(TO), low‐frequency phonon E_2_^high^, and longitudinal optical E_1_(LO) modes, respectively. The small and broad peak at 441 cm^−1^ of the E_2_^high^ mode represents oxygen motion in hexagonal ZnO. According to the literature, the decrease and broadening of this peak indicate the breaking of translational crystal symmetry due to the formation of defects or incorporation of impurities into the Zn/ZnO lattice.^[^ ^69^ ^]^ This shows that after laser irradiation, the structure of ZnO is polycrystalline with many small particles/crystallites, which correlates with the SEM images (Figure 1b). The peak at 333 cm^−1^ is attributed to E_2_^high^‐E_2_^low^, and the peak at 536 cm^−1^ corresponds to 2B_1_^low^ + 2LA. The most pronounced A_1_(LO) peak at 574 cm^−1^ indicates the presence of oxygen vacancies in the ZnO lattice and zinc interstitial defects.^[^ ^69^ ^]^ Furthermore, a broad peak at 250–300 cm^−1^ is attributed to the formation of interstitial zinc defects. These defects are formed during the processes of metallic Zn melting (during laser irradiation) and electrode surface reforming (charge/discharge).^[^ ^70^ ^]^ An additional 275 cm^−1^ peak in air‐irradiated samples corresponds to nitrogen‐related modes. During laser processing, N_2_ gas from the air is incorporated into the ZnO structure, creating a defect.^[^ ^71^ ^]^ Also, the maxima located at 284 cm^−1^ corresponds to a B_1_^low^, B_1_^high^ mode, and the maxima at 536 cm^−1^ is attributed to 2B_1_^low^ and 2LA. By using water as an irradiation medium, additional modes can be observed at 1044 to 1072 cm^−1^, referred to as TO + LO optical modes. Also, by irradiating samples in water, D and G bands of carbon appear at 1361 and 1540 cm^−1^, possibly due to dissolved CO_2_ in the water. For samples irradiated in air, an additional peak at 1158 cm^−1^ corresponding to a 2A_1_(LO), 2E_1_(LO), 2LO can be observed.

**Table 1:** Detectable Raman active modes of Zn samples.

Raman shift [cm^−1^]	Raman active modes	Indicative of	Reference
273	B_1_^low^‐B_1_^high^	Defect formation in ZnO	^[^ ^71^, ^72^, ^73^ ^]^
337	E_2_^high^‐E_2_^low^	Multi‐phonon scattering modes of ZnO	^[^ ^69^, ^73^ ^]^
382	A_1_(TO)	First‐order phonon modes of hexagonal ZnO	^[^ ^69^, ^73^ ^]^
410	E_1_(TO)	Optical phonon mode	^[^ ^73^, ^74^ ^]^
441	E_2_^high^	First‐order phonon modes of hexagonal ZnO. Oxygen vibration in ZnO.	^[^ ^69^, ^73^, ^75^ ^]^
508	E_1_(TO)+E_2_^low^	Multi‐phonon scattering modes of ZnO	^[^ ^69^, ^73^ ^]^
536	2B_1_^low^; 2LA	Overtones along L‐M and H Brillouin zone points/lines	^[^ ^73^ ^]^
574	A_1_(LO)	First‐order phonon modes of hexagonal ZnO, assigned to the formation of oxygen vacancy‐related defects.	^[^ ^69^, ^70^, ^73^ ^]^
723 – 745	LA + TO	Low‐intensity modes	^[^ ^73^ ^]^
1044 – 1072	TO + LO	Multi‐phonon scattering mode	^[^ ^69^, ^73^ ^]^
1158	2A_1_(LO); 2E_1_(LO); 2LO	Combination of second‐order LO overtones and modes	^[^ ^73^ ^]^
1361	D‐band	Disordered band of carbon	^[^ ^76^, ^77^, ^78^ ^]^
1540	G‐band	Graphitic band of carbon	^[^ ^77^, ^78^, ^79^ ^]^

The open circuit potential (OCP) measurements were also performed before electrochemical measurements (Figure 1f) to determine the cell equilibrium electrochemical potential. Freshly assembled half‐cells from samples irradiated in an air atmosphere had a predicted potential of −1.44 V versus Ag/AgCl (3 m KCl) (−1.24 V vs SHE), consistent with the reaction of Zn in alkaline media 1). Samples irradiated in aqueous media using a 266 nm laser had the same OCP. In contrast, samples irradiated in a water medium using a 1064 nm wavelength laser had a different potential and reflected a different response than expected. At the start of the measurement, the potential was −0.95 V versus Ag/AgCl (−0.75 V vs SHE) and corresponded to the reaction of a more neutral environment 2), indicating a pH change at the electrode/electrolyte interface. The concentration of OH^−^ ions should have been pH >10. However, the pH at the reaction interface was essentially pH < 10 due to ion diffusion limitations throughout the formed oxide layer on the Zn surface. Thus, the favorable equilibrium reaction (2) occurs until the imbalance is created. After some time, the electrode surface stabilizes due to electrolyte diffusion, and the concentration of OH^−^ ions increases at the interface, shifting the reaction to equilibrium (1).

(1) $$Zn{\left(OH\right)}_{2}+2e^{-}⇄Zn+2OH^{-}E^{0}=-1.245V\;vs\;SHE$$

(2) $$Zn^{2+}+2e^{-}⇄Zn\;E^{0}=-0.762V\;vs\;SHE$$

### 3.2 Electrochemical Property Analysis

**Figure 2:**
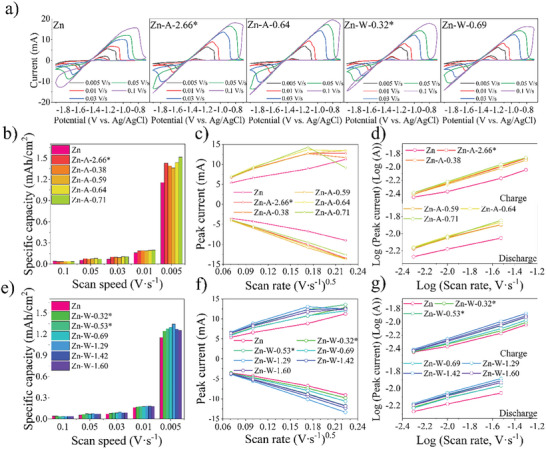
Electrochemical characterization of pure and irradiated Zn anodes: a) CV measurements at varying scan speeds from 0.005 V/s to 0.1 V/s; b) and e) specific capacities at different scan speeds; c) and f) Randles‐Sevcik graphs; d) and g) power law relationship.

Further, CV half‐cell measurements were carried out to evaluate the electrochemical properties of the irradiated sample surface. Measurements were performed with different scan speeds within a potential window of −1.9 to –0.6 V vs Ag/AgCl (3 m KCl) in an alkaline (1 m KOH) electrolyte, shown in **Figure** **2**a (measurements for all the samples are shown in Figure S3, Supporting Information). During the oxidation (anodic) sweep of all Zn samples, the surface is oxidized to the point where the electrolyte cannot reach the active Zn surface for further reaction. However, during the reduction (cathodic) sweep, a phenomenon (≈−1.3 V vs Ag/AgCl (3 m KCl)) described in the literature^[^ ^80^, ^81^ ^]^ can be observed. During a cathodic sweep in the potential range from −1.2 to −1.4 V versus Ag/AgCl (3 m KCl), an additional unreacted surface of Zn is exposed to the electrolyte, and an instant current increase can be observed due to the restored ongoing Zn oxidation process. These measurements show that the laser‐treated samples have higher oxidation and reduction currents. This suggests that a greater number of reaction centers are available on the surface, resulting in more pronounced electrochemical reactions. Also, SEM images indicate (Figure 1b) that samples have a larger surface area after the laser irradiation process due to a more detailed structure. However, the previously mentioned phenomenon (≈−1.3 V vs Ag/AgCl (3 m KCl)) is less evident in laser‐treated samples. This indicates that the reaction is more controlled, and ZnO grows more uniformly for the laser‐treated samples despite the oxidation reaction being more pronounced. Thus, suggesting that the surface laser treatment improves the overall cycling performance of the Zn electrode.

The improved cycling performance of laser‐treated samples is also evident in the specific capacity per surface area in Figure 2b,e. The results, calculated from the CV graphs, reveal that at 0.005 V s^−1^ scan rate (C‐rate of 30 C), samples that have been irradiated in an air atmosphere have a slightly larger (1.35–1.50 mAh cm^−2^) surface‐specific capacity compared to samples irradiated in a water medium (1.25–1.35 mAh cm^−2^), and even higher than a pristine Zn plate (1.15 mAh cm^−2^). For samples irradiated in air with an IR laser, increasing parameter *F* also increases the specific capacity. On the other hand, to achieve similar improved results with a UV laser, approximately twice the *F* value is required. Similar observations are made for the samples irradiated in the water medium, where increasing the parameter *F* also increases the specific capacity. However, at *F = 1.29* a maximum is reached, and a further increase of *F* leads to a decrease in capacity. Overall observations suggest that irradiated samples have an 8 – 30% surface‐specific capacity increase compared to the pristine Zn plate at 0.005 V s^−1^. The highest specific capacity from samples irradiated in the air atmosphere is Zn‐A‐0.71, and from water samples is Zn‐W‐1.29. According to the SEM images (Figure 1b), samples Zn‐A‐0.71 and Zn‐W‐1.29 are characterized by smaller average sizes and higher density of surface structures, which causes the surface‐specific capacity to increase. This is associated with a larger number of redox‐active sites where more reactions take place. Similar observations are also described in the literature for other cathode and anode materials.^[^ ^82^, ^83^ ^]^

The Randles‐Sevcik graphs (Figure 2c,f) were plotted from CV graphs and show the peak current dependency on the square root of scanning speed. As the CV graphs show (Figure 2a), Zn plating only proceeds through a partial process at faster scanning speeds. Thus, the peak current cannot be efficiently achieved and determined from the graphs. This discrepancy is also evident in Randles‐Sevcik graphs where the last point does not correlate and deviates from the others. Otherwise, the peak currents of all sample cathodic and anodic processes have a linear dependence on the square root of the scan rate. This indicates that both Zn plating and stripping are reversible and diffusion‐based processes. Similar findings can be found by analyzing the anode electrochemical kinetics with the power law relationship (Figure 2,g). It can be expressed by formula (3), where *Ip* is the peak current of anodic or cathodic reaction (mA); *v* is the scan rate (V/s); *a* represents a constant; and *b* is the power‐law exponent. Based on the value of *b*, a qualitative determination of the charge storage mechanism can be made. If *b* is 0.5, then the process is diffusion‐controlled or Faradaic, whereas if *b* is 1, it indicates that the current is surface‐controlled and the process is non‐Faradaic. The Equation (3) can be rewritten as (4); thus, by plotting *log(Ip)* versus *log(v)*, the value of *b* can be found as the slope of the graph.^[^ ^84^, ^85^, ^86^, ^87^, ^88^, ^89^ ^]^

(3) $$I_{p}=a\times v^{b}$$

(4) $$\log\left(I_{p}\right)=\log\left(a\right)+b\times \log\left(v\right)$$

The Zn sample power law plots show a linear relationship between the logarithm of peak current dependence and the logarithm of scan rate. The corresponding values of *b* are listed in **Table** **2**. The charge slopes are ≈0.5, and the discharge slopes are ≈0.3–0.4. This indicates that both cathodic and anodic processes for all samples are purely diffusion‐controlled and do not have the characteristics of a capacitor.

**Table 2:** Values of parameter *b* for all Zn samples.

Sample	Charging		Discharging	
	b value	R^2^	b value	R^2^
Zn	0.41	0.98611	0.28	0.99946
Zn‐A‐2.66*	0.53	0.99986	0.36	0.99213
Zn‐A‐0.38	0.55	0.99994	0.35	0.99376
Zn‐A‐0.59	0.51	0.99971	0.36	0.99782
Zn‐A‐0.64	0.53	0.99989	0.37	0.99611
Zn‐A‐0.71	0.53	0.99910	0.41	1.00000
Zn‐W‐0.32*	0.43	0.99555	0.32	0.99698
Zn‐W‐0.53*	0.49	0.99855	0.36	0.99997
Zn‐W‐0.69	0.46	0.99590	0.33	0.99606
Zn‐W‐1.29	0.54	0.99996	0.37	0.99803
Zn‐W‐1.42	0.52	0.99987	0.36	0.99805
Zn‐W‐1.60	0.50	0.99921	0.34	0.99911

### 3.3 Sample Characterization after Electrochemical Testing

**Figure 3:**
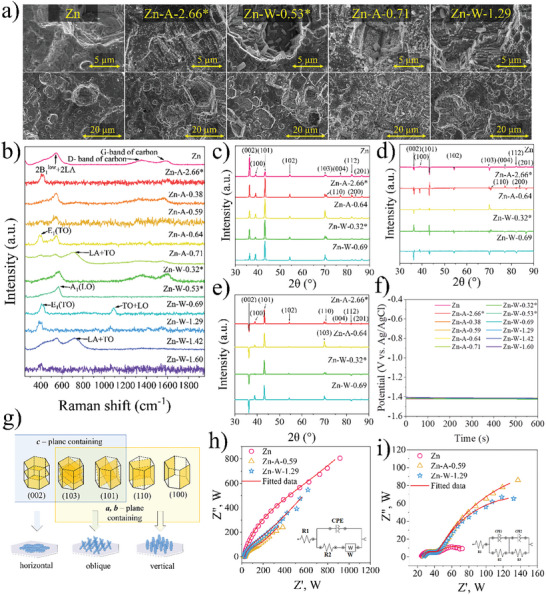
Pure and irradiated Zn sample characterization after electrochemical measurements: a) SEM images; b) Raman analysis; c) XRD diffractograms; d) difference between cycled sample XRD and their irradiated spectra (electrochemically cycled sample XRD spectra minus sample irradiated XRD spectrum); e) difference between cycled sample XRD and cycled pure Zn spectrum (CV sample XRD spectra minus CV pure Zn XRD spectrum); f) OCP; g) hexagonal Zn planes and respective growth patterns; h) EIS measurements and equivalent circuit diagrams during negative and i) positive bias.

After the electrochemical measurements, additional SEM imaging was performed to detect surface changes in the samples, as depicted in **Figure** **3**a (images for all the samples are shown in Figure S2, Supporting Information). The irradiated samples exhibited visible grain structures with growth indications on distinct surfaces of the Zn hexagonal plane. Thus, the acquired SEM pictures confirm the hexagonal Zn polycrystalline structure observed in the XRD measurements (Figure 3c). In contrast, the surface of the non‐irradiated Zn sample showed less pronounced grain structures. Almost half of the non‐irradiated sample surface displayed visible random growth signs, interspersed between large hexagonal Zn grain regions. Overall, these observations suggest that zinc laser treatment significantly enhances the plating and stripping processes, thereby improving electrochemical performance.

To gain deeper insights into surface changes during CV measurements, Raman analysis was conducted following the electrochemical measurements. The Raman spectra of irradiated samples (Figure 3b) after electrochemical measurements are noisy, and the characteristic features of ZnO are challenging to determine. In contrast, in the irradiated samples before electrochemical measurements (Figure 1f), ZnO can be clearly distinguished from pure Zn metal. After CV measurements, the non‐irradiated Zn sample showed more pronounced ZnO signals on the surface compared to the laser‐irradiated samples. This suggests that the laser treatment has enhanced electrochemical reactions, facilitating a more uniform growth of ZnO on the electrode surface and a more complete conversion back to metallic Zn. However, for the pristine Zn sample, it seems that only partial charging (conversion of ZnO to Zn) is occurring, leading to an increase in the ZnO content on the surface and consequently improving the ZnO Raman signal. This observation is consistent with the CV measurements of the pristine Zn sample, where a more pronounced phenomenon (≈−1.3 V vs Ag/AgCl (3 M KCl)) is observed, which is attributed to the formation of ZnO.

Additionally, surfaces were re‐examined using XRD measurements (Theta/2theta) to observe the changes that occurred (Figure 3c). For the non‐irradiated Zn sample, a distinct increase in (002) plane intensity and decrease in (101) plane intensity were observed, suggesting growth predominantly in a (002) plane direction. Additionally, a slight increase in the (100) plane was observed, indicating the formation of dendrite‐like or loose structures on the surface (Figure 3g). SEM images of electrochemically cycled non‐irradiated Zn sample (Figure 3a) reveal the formation of a loose structure, corresponding to the increase in the (100) plane observed in the XRD measurements, and confirming the tendency of press‐rolled Zn sheets to promote dendrite formation due to induced surface defects.^[^ ^39^ ^]^ In contrast, irradiated samples after CV measurements retained their polycrystalline (101) crystal structure formation. A slight increase in intensity was observed for the lattices containing the c‐plane, suggesting possible growth in the basal (002) plane direction of the polycrystalline surface. SEM images (Figure 3a) also confirmed more pronounced growth of crystalline structure. The slight decrease in intensities along the (100) and (200) planes may indicate that the surfaces are more resistant to vertical (dendrite‐like) structure growth (Figure 1g).

From Figure 3d, where irradiated sample spectra are subtracted from the respective electrochemically cycled sample spectra, it can be concluded that growth during electrochemical cycling mostly occurs on the surfaces and along the crystal c‐plane. The comparison of changes between electrochemically cycled irradiated samples and electrochemically cycled pure Zn anode can be seen in Figure 3e, where the spectrum of the cycled Zn anode has been subtracted from the laser‐irradiated cycled spectra. Crystal surface growth on all surfaces and planes containing the c‐plane remains dominant compared to non‐irradiated Zn samples, attributable to the chaotic crystal orientation on the surface of the Zn anode.

OCP after electrochemical testing of the samples (Figure 3f) was ≈−1.40 V vs Ag/AgCl (3 m KCl) for all the samples. This is consistent with reaction (1) indicating that there are no additional barriers for any sample that would alter the diffusion of OH^−^ ions. Further, electrochemical impedance spectroscopy was used to characterize the electrode‐electrolyte interface in the frequency range from 0.1 Hz to 100 kHz. Figure 3h shows EIS spectra at a negative DC component of −150 mV from the OCP and Figure 3i shows spectra at a positive DC component of +150 mV from the OCP. Individual EIS spectra for all the samples are shown in Figures S4 and S5 (Supporting Information). All experimental data were analyzed using NOVA 2 software, and equivalent circuits for each fitted bias are shown next to the corresponding graphs in Figures S6–S8 (Supporting Information). The obtained values of equivalent circuit components are summarized in Tables S2 and S3 (Supporting Information). In the equivalent circuit for the negative potential bias, where Zn plating takes place, the series resistance R1 for all samples is ≈27 Ω (intersection with the real resistance axis Z'). It corresponds to the ohmic resistance of the electrolyte. A semicircle follows at high frequencies, the radius of which indicates the charge transfer resistance R2. This resistance characterizes the charge transfer resistance of a faradaic process and is significantly reduced in laser‐treated samples. This resistance is ≈668 Ω for the pure Zn sample, while for the laser‐treated samples, it is ≈150 Ω. However, the CPE element, which characterizes the capacitance of the electrical double layer, increases the analog capacitance value of the laser‐treated samples. This capacitance is ≈45 µF for a pure Zn sample, while it is ≈55 µF for laser‐treated samples. This slight increase indicates a macroscopic enlargement in the surface area of the laser‐treated samples. At low frequencies, the spectra transition into a sloping tail corresponding to a Warburg impedance that characterizes diffusion‐limiting processes.

An augmented equivalent circuit model is applied to the obtained Nyquist plots at a positive potential bias, where Zn stripping and ZnO formation occur. Two semi‐circles can be seen in the Nyquist curves, indicating two parallel connections of CPE and resistance. R1, as in the previous case, describes the series resistance and is ≈27 Ω. The first parallel connection of R2 and CPE1 characterizes the condition of the electric double layer: the ion polarization resistance R2 and the electric double layer capacitance CPE1. R2 for the pure Zn sample is ≈20.5 Ω, while it is reduced to ≈17 Ω for the laser‐treated samples. The electrical double‐layer capacitance of the pure Zn sample is ≈41.4 µF and increases to ≈53 µF for the laser‐treated ones. The double‐layer capacitance values are similar to the negative biases, indicating that the laser‐treated macrosurface does not change during electrochemical cycling. The second parallel circuit of CPE2 and R3 describes a faradaic process where R3 indicates charge transfer resistance. For a pure Zn sample, this value is 38 Ω, while for laser‐treated samples, it is ≈200 Ω. This significant increase is due to the different defects formed during laser treatment, as observed in Raman spectra after electrochemical measurements (Figure 3b). This increases the charge transfer resistance in the bulk material and reduces the reaction rate for Zn stripping. CPE2 characterizes the capacitance of the Zn oxidation process and is several orders of magnitude larger than the capacitance of the double layer. It is ≈3.4 mF for a pure Zn electrode, while it is reduced to ≈2.7 mF for laser‐treated samples. This capacitance reduction is due to the slowdown of the reaction, which in turn is caused by the increased bulk material resistance.

## 4 Conclusion

In this study, the surface of Zn metal was modified using laser irradiation at two different wavelengths (266 or 1064 nm) and irradiation environments (water or air). The results indicate that the surface of the laser‐treated samples exhibits enhanced electrochemical properties. SEM images show that the surface of the modified samples has significantly more pronounced crystallinity after the electrochemical cycling compared to untreated Zn plates. This modification allows for more efficient Zn plating and dissolution during electrochemical processes. Improved Zn growth and oxidation effects were also observed in Raman spectra. It was found that laser‐irradiated surfaces had significantly less unreacted ZnO residue after the electrochemical cycling than the untreated surface.

Randles‐Sevcik plots indicated that the electrodes exhibit Faradaic characteristics typical of battery‐type electrodes, and the power law plot showed diffusion‐limited processes at the electrode/electrolyte interface. Additionally, the specific capacity of the laser‐irradiated samples was on average 8–30% higher than that of the standard Zn plate. During negative bias (Zn plating), the charge transfer resistance of the laser‐irradiated samples was significantly lower than that of the untreated Zn plate, with similar findings observed during positive bias (Zn stripping). However, in positive bias measurements, an additional semicircle with increased resistance was observed for the irradiated samples. This increased resistance can be attributed to various defects formed from air or water in the Zn plate during laser treatment, as described in references.^[^ ^90^, ^91^ ^]^

Overall, samples irradiated with a 1064 nm laser in an air atmosphere exhibited superior results compared to both untreated samples and those treated with other irradiation parameters. Additionally, the ZnO content on the Zn surface after UV irradiation at 266 nm showed no significant changes compared to IR irradiation at 1064 nm. This is attributed to the high light scattering and quantum efficiency of ZnO crystals under UV laser excitation.^[^ ^92^ ^]^ Despite the high absorption coefficient of the UV laser beam in ZnO, its power was not enough to heat and destroy the ZnO fragments. Nonetheless, the effect of the laser with suprathreshold fluence on the Zn electrode is sufficient to enhance the electrochemical properties of the system. Laser treatment increases the effective surface area and the surface‐specific capacity of the Zn electrode. This enhancement quickly reaches a saturation point with increasing laser fluence, as the surface roughness stabilizes at a certain stage of laser processing.

The overall conclusion about the fluence values used is not unambiguous, as these changes between irradiated samples are relatively small. The most noticeable changes are observed between the influence of water and air atmosphere on the irradiated sample. However, the aim of this study is to promote the understanding of the possible application of laser processing for the development of more efficient anode materials. Further experiments involving surface laser treatment are necessary to thoroughly evaluate the observed surface changes and potential improvements. Combining surface laser modification with other methods could establish a robust foundation for forming (002) basal plane surfaces in aqueous Zn‐ion batteries.

## Conflict of Interest

The authors declare no conflict of interest.

## Supporting information

Supporting Information

## Data Availability

The data that support the findings of this study are available from the corresponding author upon reasonable request.
