# Supplementary material for: Irradiating the Path to High‐Efficiency Zn‐Ion Batteries: An Electrochemical Analysis of Laser‐Modified Anodes
Source: Glob Chall. 2024 Aug 29;8(10):2400105. doi: 10.1002/gch2.202400105 (PMC11469777; doi:10.1002/gch2.202400105)
Supplement: Supplementary file 1 — Supporting Information [file GCH2-8-2400105-s001.docx]

Supporting Information

Irradiating the Path to High-Efficiency Zn-Ion Batteries: An Electrochemical Analysis of Laser-Modified Anodes

Ramona Durena*, Leonid Fedorenko, Nikita Griscenko, Martins Vanags, Liga Orlova, Pavels Onufrijevs, Sandra Stanionyte, Tadas Malinauskas and Anzelms Zukuls*

**Table S1.** Parameters of a Zn samples and visual images

| Sample name | Laser wavelength, nm | Medium | Fluence [J/cm^2^] | Image | Sample name | Laser wavelength, nm | Medium | Fluence, [J/cm^2^] | Image |
| --- | --- | --- | --- | --- | --- | --- | --- | --- | --- |
| Zn | unirradiated | – | – | 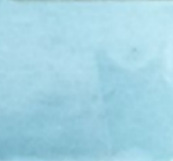 | Zn-W-0.32* | 266 | Deionized water | 0.32 | 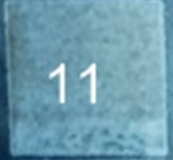 |
| Zn-A-2.66* | 266 | Air atmosphere | 2.66 | 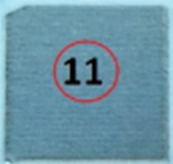 | Zn-W-0.53* | 266 | Deionized water | 0.53 | 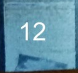 |
| Zn-A-0.38 | 1064 | Air atmosphere | 0.38 | 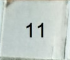 | Zn-W-0.69 | 1064 | Deionized water | 0.69 | 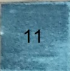 |
| Zn-A-0.59 | 1064 | Air atmosphere | 0.59 | 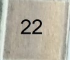 | Zn-W-1.29 | 1064 | Deionized water | 1.29 | 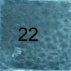 |
| Zn-A-0.64 | 1064 | Air atmosphere | 0.64 | 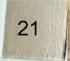 | Zn-W-1.42 | 1064 | Deionized water | 1.42 | 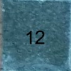 |
| Zn-A-0.71 | 1064 | Air atmosphere | 0.71 | 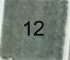 | Zn-W-1.60 | 1064 | Deionized water | 1.60 | 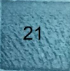 |


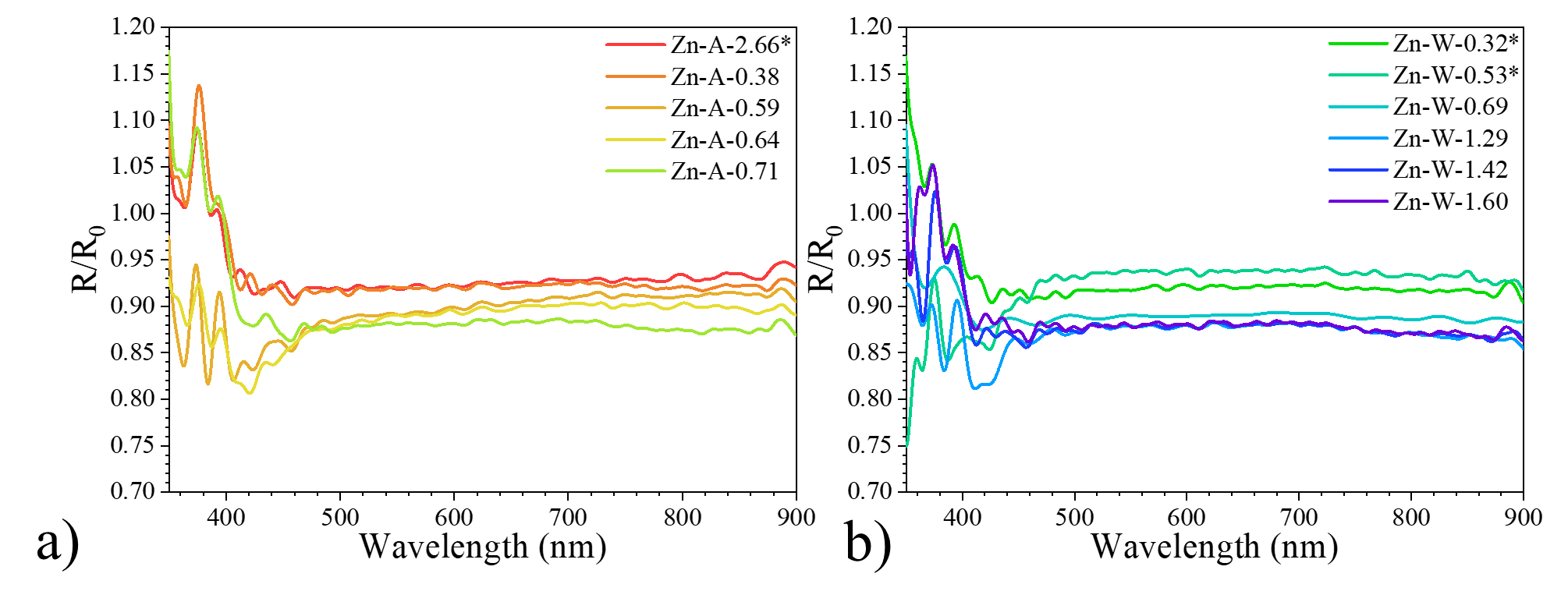


**Figure S1.** Relative diffusive reflectance of Zn sample surfaces irradiated with 266 nm and 1064 nm laser pulses in a) air and b) water medium


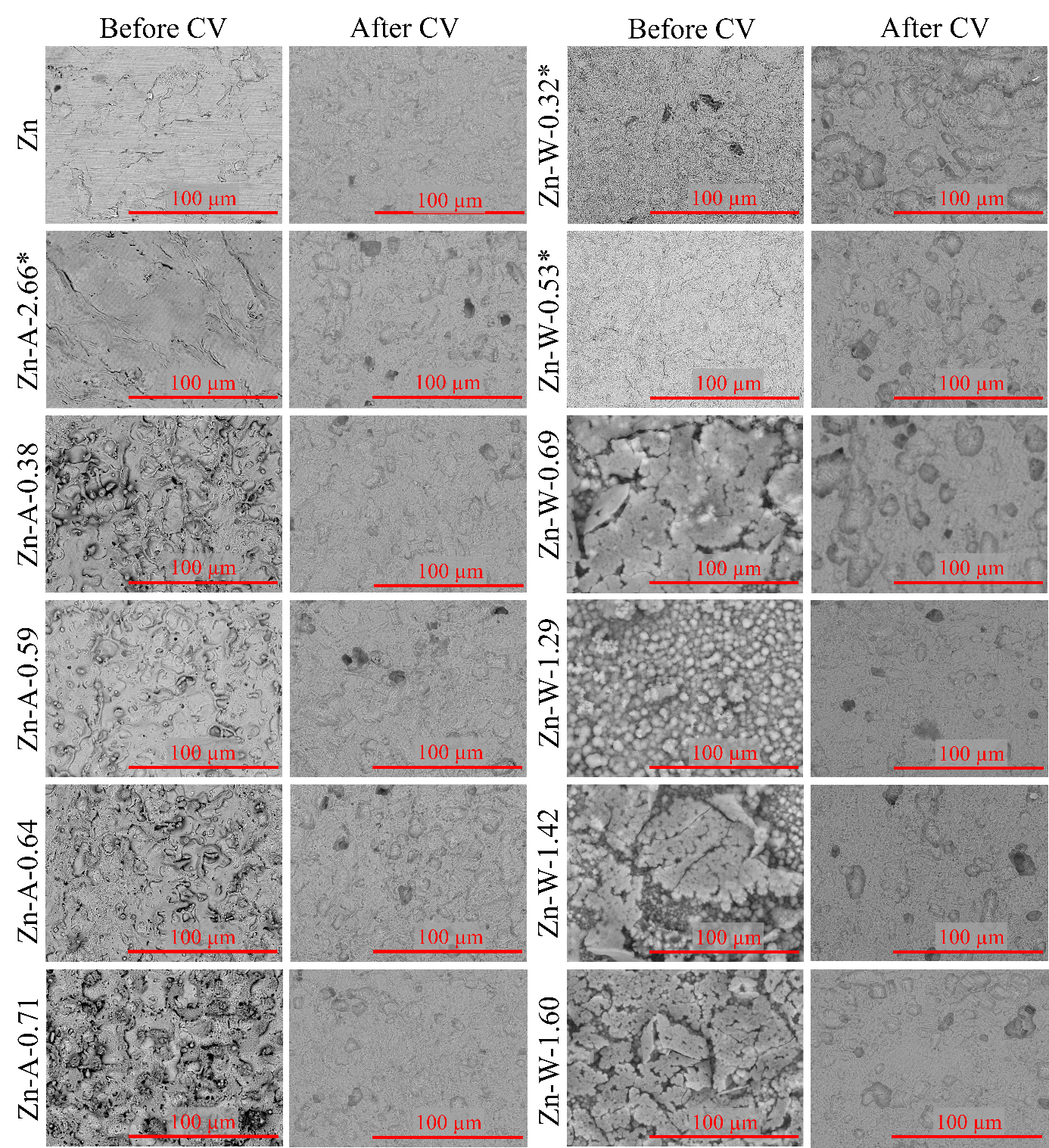


**Figure S2.** SEM images of unirradiated Zn and Zn samples irradiated in air or water atmosphere


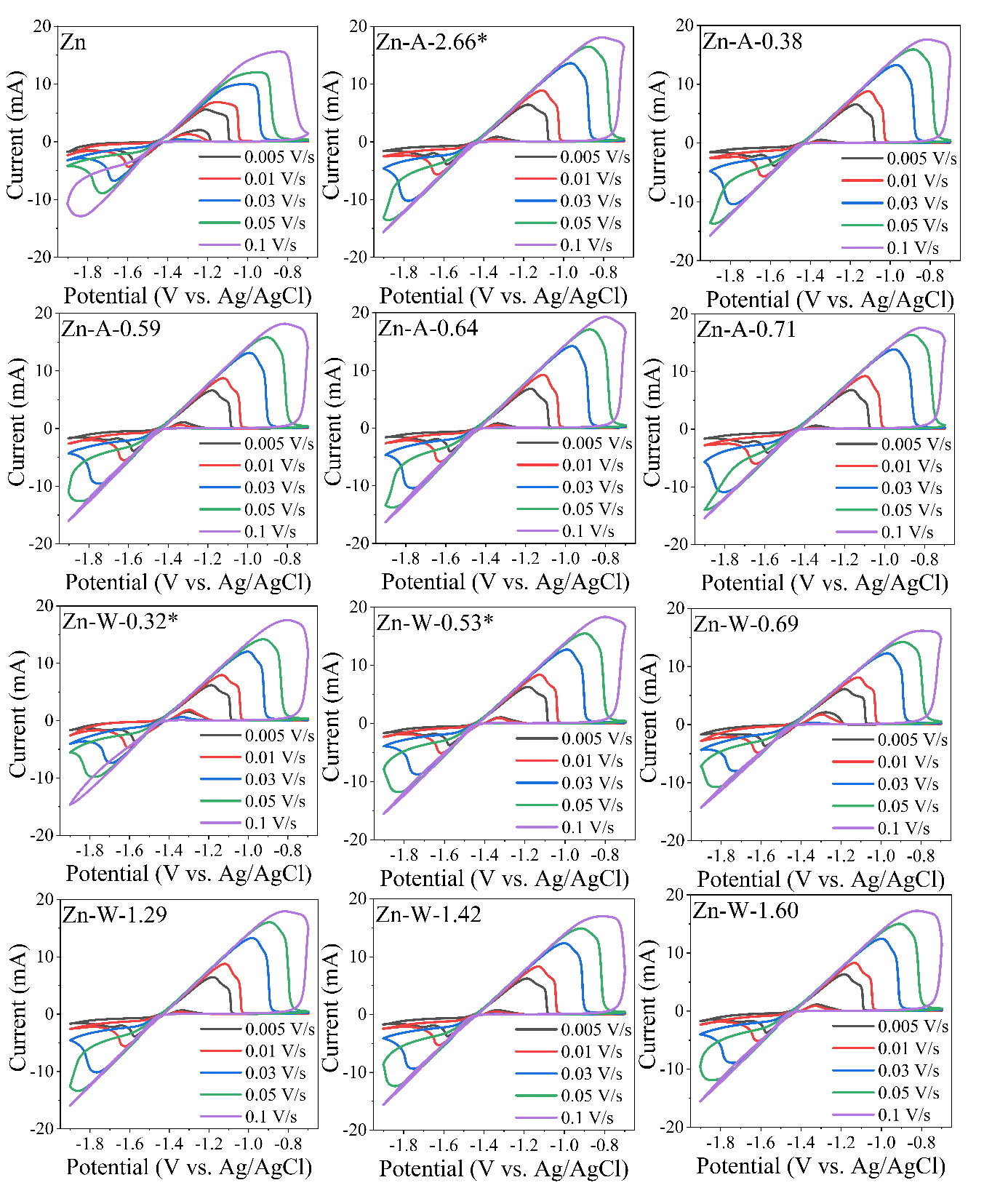


**Figure S3.** Cyclic voltammetry results of all Zn samples at different scan rates


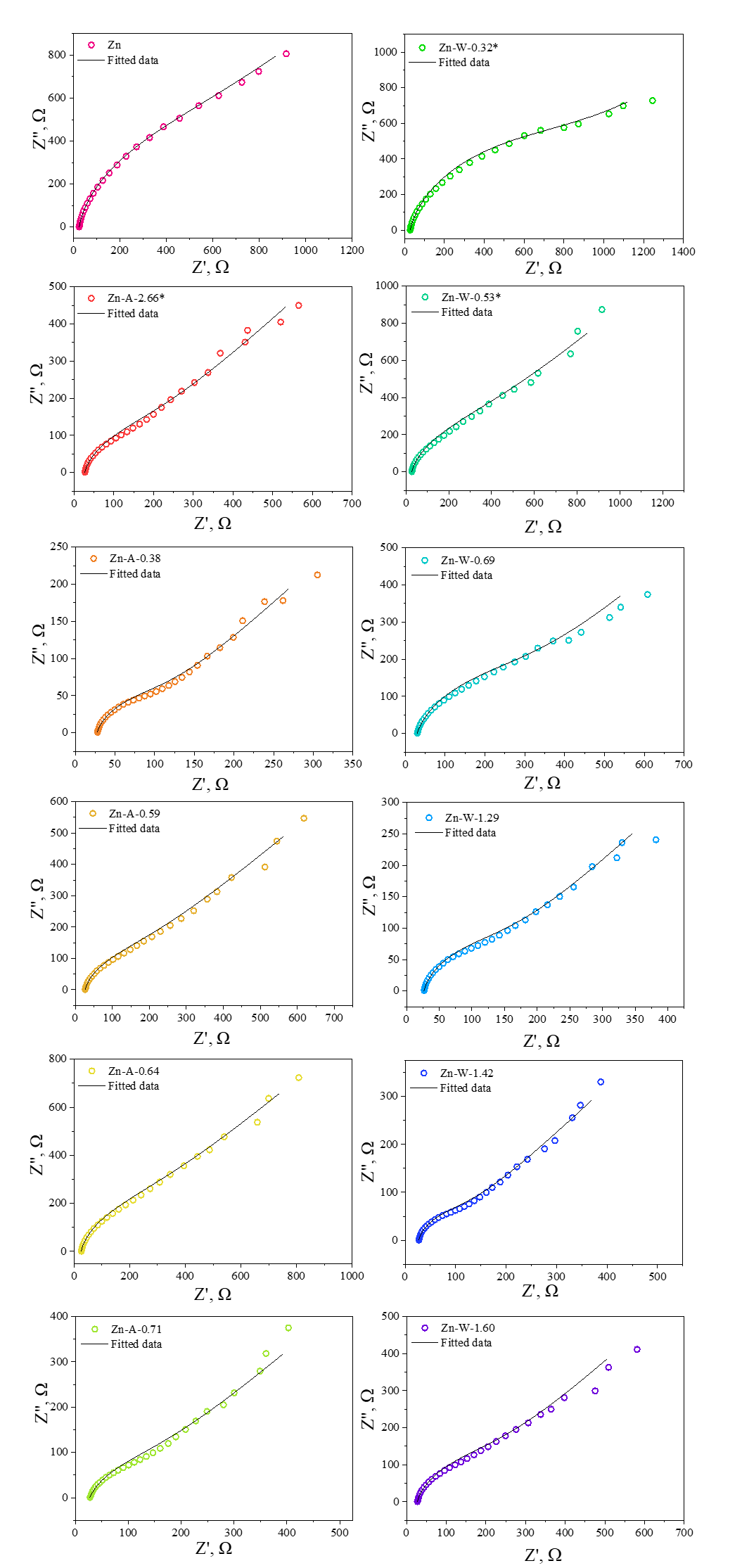


**Figure S4.** Niquist plots of all samples at negative bias


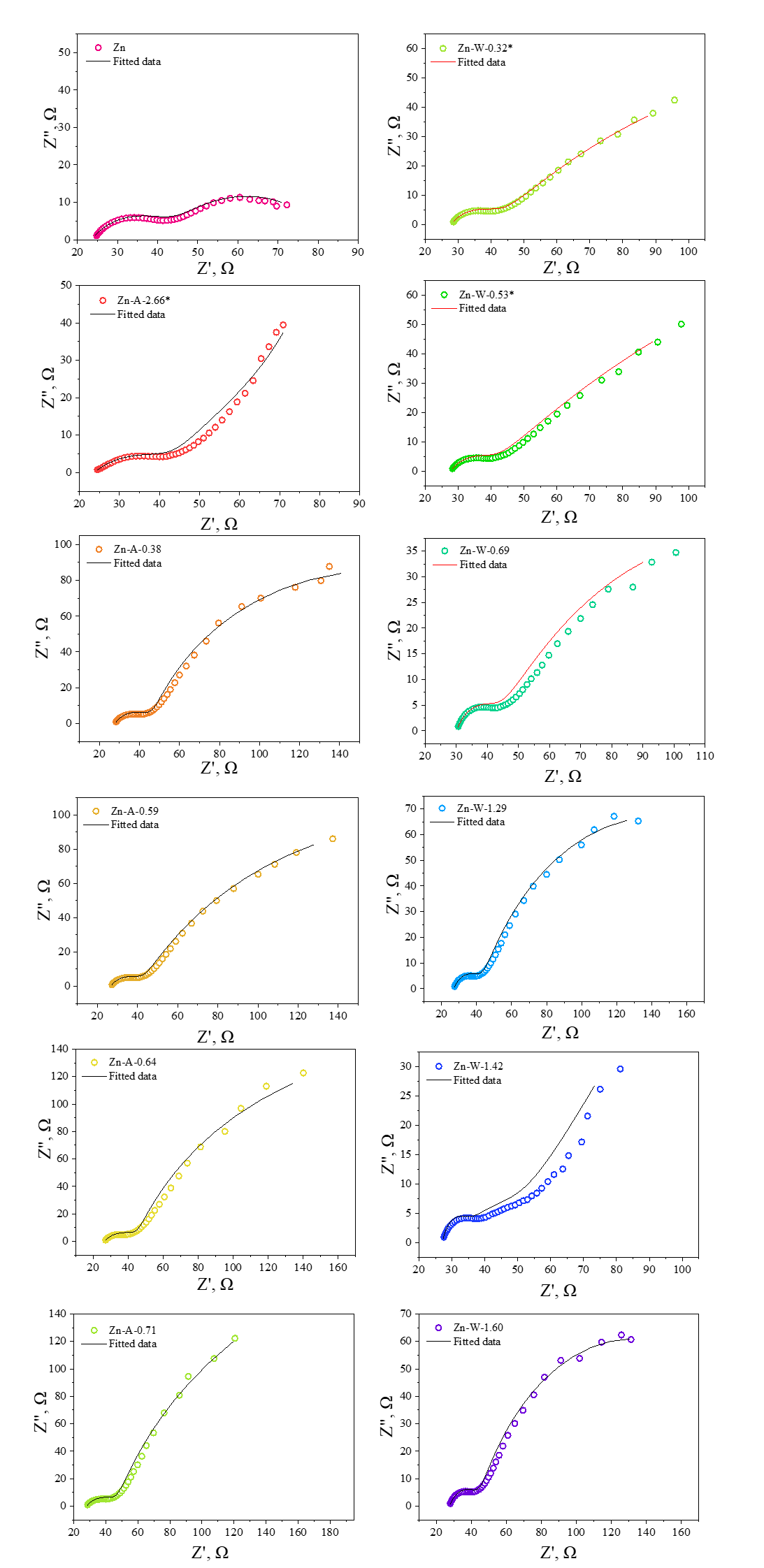


**Figure S5.** Niquist plots of all at positive bias

**Table S2.** Equivalent circuit parameters for negative bias

| Sample | R1  [Ω] | CPE | | | R2  [Ω] |
| --- | --- | --- | --- | --- | --- |
|  |  | Y_0_  [μs^N^/Ω] | N | C  [μF] |  |
| Zn | 24.7 | 69.9 | 0.90 | 45.85 | 668 |
| Zn-A-2.66* | 27.1 | 115 | 0.85 | 56.76 | 192 |
| Zn-A-0.38 | 27.5 | 156 | 0.82 | 63.70 | 91 |
| Zn-A-0.59 | 26.5 | 103 | 0.87 | 56.47 | 185 |
| Zn-A-0.64 | 26.2 | 90.1 | 0.89 | 55.96 | 292 |
| Zn-A-0.71 | 27.4 | 208 | 0.79 | 66.74 | 160 |
| Zn-W-0.32* | 27.5 | 90.3 | 0.87 | 50.26 | 912 |
| Zn-W-0.53* | 27.2 | 99.6 | 0.86 | 52.17 | 420 |
| Zn-W-0.69 | 29.3 | 151 | 0.81 | 56.87 | 310 |
| Zn-W-1.29 | 26.5 | 124 | 0.84 | 57.58 | 132 |
| Zn-W-1.42 | 26.9 | 100 | 0.86 | 52.59 | 99 |
| Zn-W-1.60 | 26.9 | 122 | 0.84 | 55.46 | 212 |


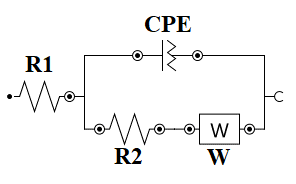


**Figure S6.** Equivalent circuit for all samples at negative bias

**Table S3**. Equivalent circuit parameters for positive bias

| Sample | R1 [Ω] | CPE1 | | | R2 [Ω] | CPE2 | | | R3 [Ω] |
| --- | --- | --- | --- | --- | --- | --- | --- | --- | --- |
|  |  | Y_0_ [μs^N^/Ω] | N | C  [μF] |  | Y_0_ [ms^N^/Ω] | N | C [mF] |  |
| Zn | 23.6 | 369 | 0.63 | 41.42 | 20.5 | 6.35 | 0.67 | 3.45 | 38.4 |
| Zn-A-2.66* | 27.5 | 384 | 0.69 | 60.70 | 11.0 | 7.60 | 0.56 | 3.11 | 250 |
| Zn-A-0.38 | 27.9 | 307 | 0.69 | 52.01 | 18.3 | 3.12 | 0.82 | 2.54 | 227 |
| Zn-A-0.59 | 26.7 | 292 | 0.70 | 53.18 | 15.5 | 3.95 | 0.72 | 2.69 | 304 |
| Zn-A-0.64 | 26.7 | 384 | 0.68 | 57.19 | 18.2 | 3.20 | 0.82 | 2.60 | 374 |
| Zn-A-0.71 | 28.2 | 386 | 0.67 | 54.08 | 18.7 | 2.98 | 0.82 | 2.45 | 506 |
| Zn-W-0.32* | 27.9 | 337 | 0.66 | 46.58 | 14.0 | 8.59 | 0.54 | 3.19 | 251 |
| Zn-W-0.53* | 27.8 | 361 | 0.70 | 61.70 | 11.9 | 8.12 | 0.54 | 3.10 | 401 |
| Zn-W-0.69 | 30.1 | 354 | 0.69 | 57.39 | 12.7 | 7.46 | 0.57 | 3.14 | 155 |
| Zn-W-1.29 | 27.0 | 290 | 0.70 | 52.93 | 16.0 | 3.79 | 0.75 | 2.72 | 200 |
| Zn-W-1.42^a)^ | 27.0 | 30.2 | 0.95 | 25.47 | 5.9 | 2.93 | 0.58 | 1.87 | 15.0 |
| Zn-W-1.60 | 27.6 | 247 | 0.71 | 49.98 | 16.6 | 3.44 | 0.77 | 2.59 | 177 |

^a)^ Equivalent circuit for sample “Zn-W-1.42” is given in **Figure S8**.


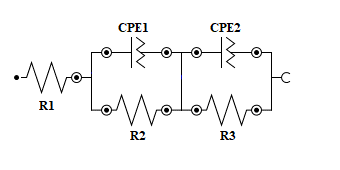


**Figure S7.** Equivalent circuit for all samples at positive bias


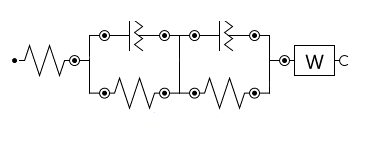


**Figure S8.** Equivalent circuit for sample Zn-W-1.42
